# Antiasthmatic Medicinal Plants of Tanzania: An Ethnomedicinal and Ethnopharmacological Review

**DOI:** 10.1155/2024/4420431

**Published:** 2024-08-27

**Authors:** David Sylvester Kacholi

**Affiliations:** Department of Biological Sciences Dar es Salaam University College of Education University of Dar es Salaam, P.O. Box 2329, Dar es Salaam, Tanzania

## Abstract

Traditional medicinal plants (TMPs) are a significant part of people's quality of life, offering a natural substitute for modern drugs with numerous side effects. In Tanzania, data on antiasthmatic TMPs are highly fragmented. This review, a comprehensive compilation of ethnobotanical research evidence, aimed to provide a thorough understanding of TMPs used by the locals for asthma management and identify species that have already been investigated in preclinical studies. The review was conducted according to the Preferred Reporting Items for Systematic Reviews and Meta-Analyses (PRISMA) statement. To gather relevant literature on antiasthmatic TMPs used by Tanzanians, a web search using electronic databases (Scopus, PubMed, ProQuest, Academic Library, Web of Science, SciFinder, Wiley Online Library, Google Scholar, ScienceDirect, and African Journals Online) was conducted. The scientific names were verified through the Plants of the World Online database, and the collected information was analysed for descriptive statistics using Microsoft Excel software. The ethnomedicinal information was obtained from 24 different articles. Microsoft Excel software was used to analyse the data using descriptive statistics. A total of 62 TMPs belonging to 33 families were identified. Species of the Fabaceae (14.5%) and Rubiaceae families (8.1%) are the most utilized. The analysis revealed that trees (42.0%) and leaves (40.0%) are the most utilized life forms and plant parts, respectively. Most plant materials (59.7%) used to make remedies were collected from the wild environment. Decoction (55.0%) is the dominant preparation method of remedies, and the majority (69.0%) were orally administered. Of the recorded TMPs, 22.6% had their in vivo antiasthmatic activity reported in the literature. The review also highlighted the strategic significance of preparations of remedies made from TMPs for discovering and developing new antiasthmatic drugs. However, the need to identify the molecular targets of action and toxicological aspects of the TMPs should be considered.

## 1. Introduction

Asthma is a life-threatening respiratory disorder affecting people of all ages worldwide. The disorder is caused by inflammation and muscle constriction around the airways, resulting in clinical symptoms such as chest tightness/pain, breathing difficulties, triggering coughing, a whistling sound (wheezing) when exhaling, and shortness of breath [1, 2]. Over the past decades, the prevalence of the disorder has increased progressively, and it is estimated that more than 100 million people may be affected by 2025 [3]. According to the World Health Organization (WHO), about 455,000 deaths worldwide were recorded in 2017 to be caused by asthma. The disorder remains the most frequent noncommunicable disease (NCD) in children and adolescents in most African countries. With NCDs set to surpass infectious diseases in the continent by 2030, efforts to address asthma are required. Despite this, asthma often remains undiagnosed and is associated with considerable morbidity and mortality. Also, environmental exposure, stigma, and poverty worsen outcomes for a population with asthma [4]. The prevalence of asthma in developing and developed countries varies greatly from less than 5% to about 20%, respectively [5]. The pervasiveness of the disease has been growing over the past decades, with the highest increase seen among children and young adults. In Tanzania, the prevalence of the disease condition, such as wheezing, is reported to be between 12.1% and 23.1%, while that of exercise-induced asthma is between 2.4% and 26.3%. On the other hand, self-reported asthma is between 6.4% and 17.6% [6].

Traditional medicinal plants (TMPs) are a perpetual and treasured source of novel bioactive agents. TMPs' secondary metabolites, such as flavonoids and phenolic compounds like apigenin, quercetin, kaempferol, rutin, mangiferin, and luteolin, have been investigated and found to prevent and treat respiratory disorders, including asthma [7–11]. Nevertheless, despite the pharmacological and therapeutic potential of compounds of TMP origin, documentation of medicinal plants in Tanzania has not been adequately done, and registration of patents has declined in the last decades. Thus, such circumstances are worrisome and divulge the need for actions to increase the research on natural products, especially given Tanzania's rich biological diversity of plants [12, 13]. The locals in Tanzania have been using diverse TMPs such as *Mangifera indica* L. [14, 15], *Ocimum basilicum* L. [16], *Leonotis nepetifolia* (L.) R.Br. [17, 18], *Balanites aegyptiaca* (L.) Delile [19, 20], and many others to treat asthma. These TMPs can be considered important sources of bioactive compounds that can be used in the formulation of novel drugs for asthma management.

It is apparent that TMPs significantly enhance people's health and quality of life and that Tanzanian biodiversity is vital in the search for new bioactive ingredients with promising effects against asthma. Thus, the present review aims to offer a comprehensive list of the scientific records on TMPs used to manage asthma in Tanzania and identify the TMPs already studied in preclinical and clinical assays. This review is the first in the country to bring together all the records in the literature on the TMPs of Tanzania flora used to manage asthma.

## 2. Methods

### 2.1. Literature Search Strategy

**Figure 1:**
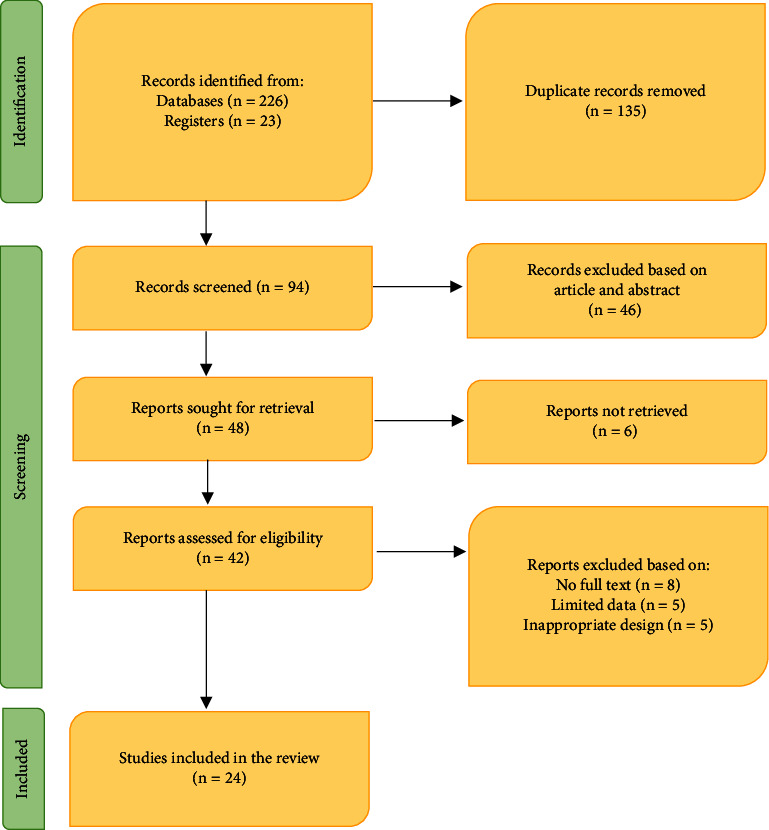
PRISMA flow diagram of the study.

This comprehensive review has gathered data on antiasthmatic TMPs used by Tanzanian communities. The review was accompanied by the Preferred Reporting Items for Systematic Reviews and Meta-Analyses (PRISMA) statement (Figure 1) [21]. Scopus, Medline (PubMed), ProQuest, Academic Library, Web of Science, SciFinder, Wiley Online Library, Google Scholar, ScienceDirect, African Journals Online, and OpenGrey were systematically searched for relevant published peer-reviewed articles, books, and reports related to antiasthmatic TMPs. The following keywords: “traditional medicinal plants,” “herbal/traditional remedies/medicines/therapies,” “ethnobotany,” “ethnomedicine,” “ethnopharmacology,” “medicinal plant,” and “phytomedicine,” were searched in combination with keywords regarding “asthma,” “anti-asthma,” “anti-asthmatic,” “United Republic of Tanzania,” and “Tanzania.” The accuracy of the identified TMPs' scientific names was verified through the Plants of the World Online (https://powo.science.kew.org/) botanical database. Also, each recorded TMP was searched using the same databases to obtain information on their antiasthmatic potential and safety. Thus, all articles reporting antiasthmatic activities and the safety of the TMPs were surveyed, and their findings were summarized. Ethnomedicinal uses of the recorded TMPs in other African countries were recorded, too.

### 2.2. Eligibility Criteria

All peer-reviewed articles, book chapters, conference publications, and books presented in the English language were included. Still, oral presentations, review articles, hypotheses, letters, nonretrievable articles, articles with limited TMPs data, and those not well designed were excluded. Moreover, all studies that had different attributes such as family name, local plant name, habitat, life form, plant part used, preparation mode, and application route were included in the study.

### 2.3. Ethnomedicinal Data Analysis

The collected ethnomedicinal information was entered into the Microsoft Excel software and analysed for descriptive statistics of the recorded botanical families, TMPs, plant parts used, life forms, methods of preparation, routes of application, and associated indigenous knowledge. The findings were then presented as tables and figures.

## 3. Results and Discussion

### 3.1. Literature Search

A total of 249 potential studies were included in the database. The exclusion of duplicates led to the initial screening of 94 articles based on their titles and abstracts. After initial screening, 46 articles were eliminated, and 48 were subjected to screening based on retrievability, where six articles were excluded as they were not retrievable. Then, the 42 retrievable articles were screened for eligibility, and only 24 studies were included in this systematic review. Figure 1 displays the PRISMA flowchart and selection process for the review.

### 3.2. Distribution of Antiasthmatic Traditional Medicinal Plants

**Figure 2:**
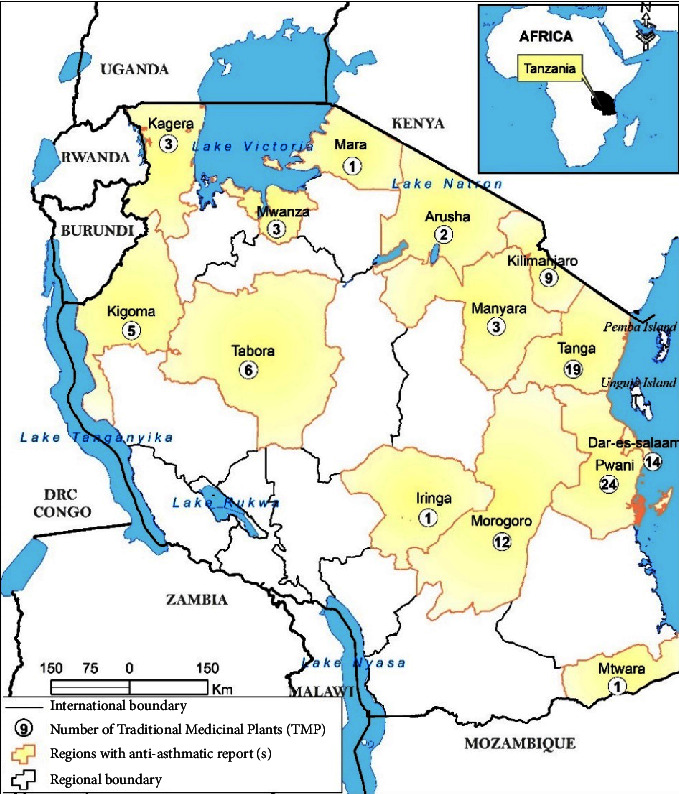
Map showing distribution and number of antiasthmatic traditional medicinal plants (TMPs) in Tanzania.

This review has recorded 24 ethnobotanical studies presenting TMPs from 14 regions out of 31 administrative regions of Tanzania (Figure 2). The findings indicate that research on the prevalence of asthma in Tanzania is limited. The majority of the recorded antiasthmatic TMPs were reported in the Pwani Region (37.8%), followed by the Tanga Region (30.6%), Dar es Salaam (22.6%), Morogoro (19.4%), Kilimanjaro (14.5%), and Tabora (9.7%). Arusha (3.2%), Mtwara, Mara, and Iringa (1.6% each) regions had fewer antiasthmatic plants (Figure 2). The high number of reported antiasthmatic TMPs in the Pwani, Tanga, and Dar es Salaam regions indicates that the locals in the regions have good aboriginal knowledge on using TMPs against asthma, and possibly these regions have many asthmatic incidences.

### 3.3. Diversity of Antiasthmatic Medicinal Plants

This review identified sixty-two (62) TMPs belonging to 33 families used by Tanzanian communities to treat asthma. The most commonly used TMPs belonged to the family Fabaceae (9 species, 14.5%), followed by Rubiaceae (5 species, 8.1%), Euphorbiaceae, Lamiaceae, and Rutaceae (4 species, 6.5% each), and Anacardiaceae and Capparaceae (3 species, 4.8% each) families (Table 1). The predominant use of these families for treating asthma and related complications is attributed to possessing a widespread range of bioactive ingredients, making them primarily effective in managing human ailments [31, 67]. As in many African countries, the general use of TMPs for treating asthma and other ailments among Tanzanians is due to their affordability, cultural acceptability, ease of access, and fewer side effects [71–73]. This suggests that TMPs are vital alternatives to the presently accessible conventional asthma remedies, especially for low-income families. Similar to this study's findings, other ethnobotanical studies conducted in Brazil [31], India [74], South Africa [22], Togo [23], and Rwanda [32] have also reported the dominant use of antiasthmatic TMPs belonging to Fabaceae. Euphorbiaceae [24], Rutaceae [32], and Lamiaceae [75] are extensively used to treat several allergic inflammatory conditions, including asthma. Additionally, the prevalent and high utilization of TMPs from these botanical families indicates that they are broadly distributed all over the globe.

### 3.4. Plant Life Forms, Habitats, and Parts Used

Medicinal plants identified in this review were mainly trees (43.6%), followed by shrubs (30.6%) and herbs (25.8%). This observation, however, is not surprising since these life forms are dominant components of local flora across the country. The more familiar the TMPs' life form is in an area, the greater the likelihood of its prevalent utilization is [22, 76]. Therefore, the locals in Tanzania might wish for the aforesaid life forms due to their abundance, ease of accessibility, and familiarity. Most collected TMP resources were from the wild environments (59.7%), followed by wild and cultivated environments such as farms and home gardens (21.0%) and cultivated areas (19.3%). A similar finding was reported in Ethiopia [77, 78], Cameroon [79], and South Africa [80], whereby most herbal medications were collected from wild. The dependency on wild resources is because they are free, as no licences are obligatory for collection. TMP users consider cultivated plant resources less effective than wild ones [42]. Hence, conserving and protecting wild environments is paramount for ensuring a sustainable supply of TMPs. Moreover, the study suggests the need to adopt both macro and micropropagation strategies as a conservation strategy and an excellent means of reducing the exploitation pressure on wild resources. The strategy will ensure future access to the remedies of TMPs and provide a sustainable supply of raw materials for developing affordable modern drugs through up-to-date science.

**Figure 3:**
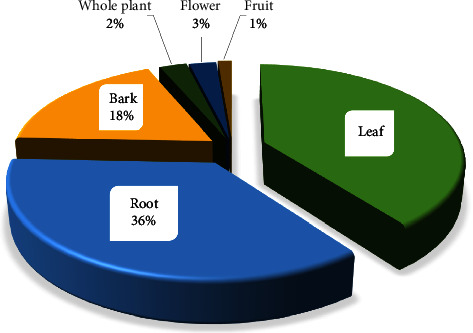
Percent share of plant parts used for making antiasthmatic herbal remedies.

It was observed that leaf (40%) was the most preferred plant part for antiasthmatic remedy preparation, followed by root (36%), bark (18%), flower (3%), whole plant (2%), and fruit (1%) (Figure 3). The high use of leaves compared to other TMPs parts might be due to their effectiveness, which is related to a higher accumulation of bioactive ingredients with therapeutic potential, ease of harvest, and swift ability to regenerate [81]. The TMP leaves are also known for synthesising most secondary metabolites that are beneficial in therapeutic activities [82]. The widespread use of leaves for asthma management corroborates with the findings reported in Brazil [31] and Togo [23], which also reported the highest use of leaves for asthma treatment than other plant parts. In contrast to this study's finding, the root was reported to be commonly used to treat asthma and related conditions in South Africa [22]. Despite being rich in bioactive compounds, the frequent harvest of roots can endanger the survival of TMPs [82]. Therefore, the use of leaves is highly encouraged if they can be used to serve the same purpose. Appropriate harvesting tactics and conservation strategies are crucial for safeguarding the sustainable utilization of TMPs resources.

### 3.5. Preparation and Administration

**Figure 4:**
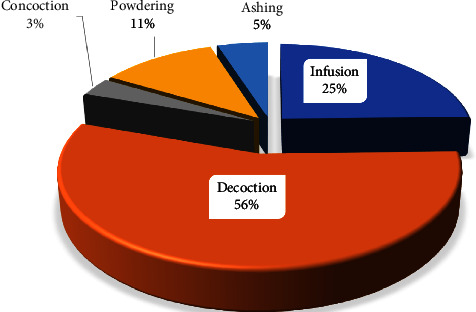
Mode of preparation of antiasthmatic remedies.

Decoction (56%) and infusion (25%) were the most common methods of preparing antiasthmatic remedies. Other methods include powdering, ashing, and concoction (Figure 4). Decoctions involve boiling collected TMPs materials in a specific quantity of water and allowing the mixtures to cool before administration. In contrast, infusion involves pouring hot or warm water onto the plant material and allowing the mixture to cool before administration. The high preferential use of decoction in preparing remedies is due to its simplicity of preparations, and it helps the extraction of bioactive compounds and preservation of the herbal remedies longer than cold water [83]. Moreover, the decocts are swiftly absorbed and have considerable actions in all traditional types of practice [84]. Various studies have also reported decoction [81, 85–87] as the foremost method for preparing herbal remedies for treating various human ailments. Furthermore, 66% of the preparations of antiasthmatic remedies involve using water as a solvent. Water is preferred because of its affordability and efficiency during extraction [88]. Other additives such as soda ash, honey, tea leaves, porridge, and juices are usually used to increase the potency of the therapies, making the preparation pleasant and evading intestinal distress [89]. The oral route (69%) was reported to be the main pathway for administering antiasthmatic remedies. A similar finding was also reported in Brazil [31], Togo [23], and South Africa [22].

### 3.6. Similar Traditional Use of Medicinal Plants Elsewhere

Moreover, the review has revealed that 59.7% of the recorded TMPs are similarly used for asthma management in other parts of the world (Table 1). For instance, *Euphorbia hirta* L. (Euphorbiaceae), *Abrus precatorius* L. (Fabaceae), and *Moringa oleifera* Lam. (Moringaceae) were reported in Togo and Nigeria [23, 24]. Other antiasthmatic TMPs recorded elsewhere include *Ocimum bacilicum* L. (Lamiaceae), *Leonotis nepetifolia* (L.) R.Br. (Lamiaceae) *Musa paradisiaca* L. (Musaceae) are as antiasthmatic in Brazil [31], *Ziziphus mucronate Willd.* (Rhamnaceae), *Osyris lanceolata* Hochst. and Steud. (Santalaceae), and *Withania somnifera* (L.) Duna (Solanaceae) in South Africa [22], *Harrisonia abyssinica* Oliv. (Rutaceae), *A. precatorius* in Kenya [51], *W. somnifera* in Ethiopia [67], and *Dysphania ambrosioides* (L.) Mosyakin and Clemants in Bolivia [28] and Morocco [27]. The wide utilization of TMPs by different ethnic groups from other countries indicates that TMPs possess potential antiasthmatic bioactive ingredients that can be used to develop affordable modern drugs.

### 3.7. In Vivo Antiasthmatic Activities of Some Medicinal Plants Reported in Tanzania

Out of the total recorded TMPs in this study, only 22.6% (14 TMPs) have been examined for their antiasthmatic potential through preclinical investigations using experimental animal models (Table 2). The dominant animal models used were mice, guinea pigs, and Wistar rats. The pharmacological potential of the TMPs was assessed using extracts, essential oil, fractions collected from the extracts, and isolated compounds. The ovalbumin-induced (OVA-sensitized) asthma model was the most preferred one for evaluating the pharmacological effect of the products obtained from TMPs. The reported information on *in vivo* antiasthmatic activities of the TMPs (Table 2) is of great significance in supporting or refuting the indications of the use of the recorded TMPs in traditional medicine. For instance, fresh seeds of *O. basilicum* were found to have immunomodulatory and anti-inflammatory effects and tended to lessen mucus hypersecretion in mice's OVA-induced allergic asthma model [104, 107]. The ethanol extract of *A. precatorius* leaves at doses of 100, 125, and 150 mg/kg was evaluated and revealed to inhibit clonidine-induced catalepsy significantly and possess antihistaminic activity [90]. Moreover, the n-butanolic fraction (NBF) of the fruit pulp of *Balanites aegyptiaca* (L.) Delile showed a dose-dependent (at 50, 100, and 200 mg/kg p.o.) beneficial effect on the degranulation rate of actively and passively sensitized mesenteric mast cells of albino rats when challenged with antigen (horse serum). Also, the NBF fraction significantly reduced the serum IgE level and number of eosinophil cell count in rats, showed significant antihistaminic activity in histamine-induced contraction in goat tracheal chain preparation, and resulted in substantial protection against acetylcholine and histamine aerosol-induced bronchospasm in guinea pigs [70].

Among the recorded TMPs used for asthma management in this review, only two, *Mangifera indica* L. and *M. paradisiaca*, have had their antiasthmatic potential investigated through randomized clinical trials. The species revealed relevant antiasthmatic activity, and their products can be used as an effective alternative to complementary therapy for asthma. These results are essential to validate the indications of Tanzanian communities' traditional use of these plants [31]. On the other hand, the clinical trials of the pseudostem powder capsules of *M. paradisiaca* showed no therapeutic effects when administered to asthmatic patients [108], while *Acalypha fruticosa* Forssk possesses powerful bronchodilatory, anti-inflammatory, antihistaminic, antiallergic, and mast cell stabilizing activities [91]. These plants proved to be efficient antiasthmatic agents and, hence, can be used to discover modern drugs.

### 3.8. Future Research and Perspectives

This review disclosed that locals in Tanzania rely on TMPs to treat various human ailments, including asthma, and are well informed about their identities and use of the TMPs. Data collected in this review demonstrate that asthma is treated with a good number of TMPs. The present findings correlate with ethnobotanical studies conducted in various parts of Africa, such as South Africa [22], Togo [23], and Nigeria [24], as well as those conducted elsewhere in the world, such as in India [74] and Brazil [23], that asthma is a life-threatening disorder globally. Reports of comparable therapeutic applications of the documented TMPs in Africa and the rest of the world (Table 1) show that these TMPs are treasured sources of ethnomedicines. The comparative analysis reinforces the firm belief that indigenous knowledge represents an important heritage developed over the centuries and a considerable mass of data that should be exploited to provide new and valuable knowledge on plant resources. Therefore, it is essential to preserve this knowledge through proper documentation, plant species identification, herbal preparation, and dosage. The present review will help future research on the choice of TMPs to investigate phytochemical safety and pharmaceutical efficacy. Furthermore, there is a need for more research on the bioactive compounds of these TMPs, some of which have already revealed antiasthmatic activities, as shown in Table 2. Also, the study suggests a need to establish the linkage between the bioactivity and specific compounds responsible for the extensive use of these TMPs. The documented indigenous knowledge in Tanzania and available scientific literature sturdily suggest that at least some of the TMPs used as medications can be potential sources of new contemporary drugs.

Currently, phytochemical and pharmacological analysis of TMPs is vital in medicinal plant research and indigenous knowledge systems. Thus, sharing such knowledge is central to maintaining possibilities for using TMPs, particularly as alternative herbal medicine is mounting because of its low costs and increasing belief in herbal remedies. Worldwide, knowledge of orthodox pharmaceuticals has originated from traditional indigenous knowledge. For instance, many of the conventional drugs traded in today's market have a long history of applications as traditional medications; among them are quinine, opium, and aspirin. While Tanzania is gifted with a robust culture of traditional medicine usage for primary healthcare, there is a need to normalize the preparation methods, dosage, and route of administration. Validating the relationships of the ethnomedicinal uses, bioactive compounds, and biological and pharmacological effects is highly significant and is still a primary task for future investigations. Moreover, efforts are required to explore the physiological and biochemical activities demonstrated by the recorded TMPs, and identification of the bioactive ingredients and their associated mechanisms of action is paramount.

Like many other African countries, Tanzania is an imperative repository of TMP applications in primary healthcare. This is echoed in the variety of TMPs used for therapeutic purposes in various regions in the country and the extensive range of their usages and associated indigenous medicine procedures [12–20, 76, 86]. The country's demand for traditional medicines to cure various human ailments has increased enormously. As the market continues to accelerate, awareness should be created among local communities to ensure sustainable use and conservation of the TMPs. A collaborative approach to sustainable use, conservation, and management of TMPs should be implemented, and all stakeholders should be involved. Communities in Tanzania should be actively engaged in managing plant resources as they rely on them for their primary healthcare needs. Hopefully, this will balance meeting their health needs and wise utilization of plant resources to ensure sustainable development. Like other forms of biodiversity, the severe threats to TMPs are habitat loss and fragmentation, climate change, population pressure, and invasive species. The study suggests that the conservation of TMPs should be promoted through cultural beliefs and norms, government laws and policies, the development of guidelines for the use of TMPs, the incorporation of local traditional cultural values in the national biodiversity conservation agenda, and their cultivation on farms and home gardens to overcome declining supplies and the risk of extinction from natural sources. So far, it is not well known whether overexploitation of TMPs is an issue in Tanzania. However, future ethnobotanical studies should also focus on how locals use and manage TMPs. Such studies will offer an understanding of how locals in the country relate to the plant resources they use for therapeutic purposes.

### 3.9. Challenges of Integrating Traditional Medicine into Modern Health Systems in Tanzania

The WHO recognizes the important role of traditional medicine, including the TMPs, and endorses its integration into the healthcare system. In sub-Saharan Africa, nearly 85% of the population relies on traditional practitioners for their primary healthcare needs due to limited access to modern facilities [86]. It should be noted that integrating traditional medicine into national health systems can increase access to primary health care for many people who only seek the services of traditional health practitioners [109]. Integration can be predominantly beneficial in dealing with health crises such as pandemic outbreaks. Regardless of the opportunities and efforts done by the government of Tanzania, the integration of traditional medicine into modern healthcare systems still faces several challenges, not limited to those outlined below.

#### 3.9.1. Absence of Essential Resources and Scientific Validation

Notwithstanding the growing national recognition of traditional medicine in the country, its incorporation is hindered by the lack of necessary resources and operational policies to expedite integration. While an appreciable proportion of traditional medicines is deeply rooted in cultural and spiritual practices, adequate scientific validation is lacking. Additionally, the fundamental resources for research to address the challenge are insufficient.

#### 3.9.2. Inadequacy of Well-Trained Traditional Health Practitioners

The scarcity of well-trained traditional health practitioners (THPs) to advocate and pioneer the integration of traditional medicine into systematic health care is one offshoot of the aforementioned challenges. Conventional medicine is primarily informal and does not require formal education or professional training as it passes from generation to generation orally. Therefore, it is susceptible to fraud and dishonesty, and practitioners' unproven medical claims are encouraged to thrive. These features of the practice incite a lack of trust in traditional medicine among the public and modern medicine practitioners.

#### 3.9.3. There Are No Regulatory Frameworks for Quality Control

Ensuring the quality, safety, and efficacy of herbal medicines and associated services is fundamental.

#### 3.9.4. Potential Conflict between Traditional and Modern Medical Practices

For example, while the country has made efforts to integrate conventional and complementary medicine into its health system, regulating the subsector has remained a challenge due to the lack of information and operational factors facing the regulatory frameworks in the country.

#### 3.9.5. Knowledge Transfer

The transmission of knowledge and ethical utilization of intellectual property are vital to the growth and development of science. Nonetheless, most traditional medicine practitioners neither practice proper documentation nor observe a systematic knowledge-sharing approach. Instead, knowledge is commonly shared orally with some individuals in the conventional medicine field. This transfer of knowledge is done casually, often spanning years, and potentially contributes to knowledge loss, given the possibility of death. Furthermore, the fear of creating competition sometimes surpasses the desire to provide care, leading to knowledge hoarding. Also, the unethical utilization of traditional medicine knowledge gained from traditional practitioners by researchers without appropriate acknowledgment is another reason for the increased hesitancy of some traditional health practitioners to share their knowledge.

## 4. Conclusion

Since time immemorial, TMPs have been used to manage different human ailments, including asthma. In Tanzania, sixty-two TMPs are being used to treat asthma. Most of the recorded TMPs belong to the families Fabaceae and Rubiaceae. Trees and leaves are the most utilized life forms and plant parts, respectively, and most plant materials were collected from the wild areas. Decoction is the principal method of preparing remedies, and most herbal remedies are administered orally. Consideration must be given to the sustainable harvest of these TMPs so that future generations can enjoy what others have already enjoyed from Mother Nature. Also, given the significant number of TMPs that have not been scientifically studied, pharmacological research is needed to identify the bioactive ingredients responsible for the therapeutic effects connected with the preparations' use, and safety/toxicological studies involving the herbal remedies are obligatory, too. Also, in view of the popularity of the listed TMPs, there is a need to design clinically relevant randomized clinical trials for herbal remedies in the management of asthma. Generally, this systematic review discloses the TMPs used by the locals in Tanzania against asthma and provides an entryway for future studies to discover and develop new modern drugs to manage asthma.

## Figures and Tables

**Table 1:** Medicinal plants used traditionally to manage asthma in Tanzania.

Family	Species name	Vernacular name (region)	So	LF	PU	MoP and MoA	References	Similar use in other countries
Alliaceae	*Allium sativum* L.	Kitunguu maji (Morogoro)	C	H	L	Concoction drunk	[14]	South Africa [22], Togo [23], Nigeria [24, 25]

Amaranthaceae	*Chenopodium ambrosioides* L. Syn: *Dysphania ambrosioides* (L.) Mosyakin & Clemants	Mdiwasoko (Kilimanjaro)	W, C	H	Whp	ns	[26]	Morocco [27], Bolivia [28]

Anacardiaceae	*Lannea schweinfurthii* var. *stuhlmannii* (Engl.)	Mumbu (Tanga), Sayu (Mwanza) Mtundutwa (Kilimanjaro)	W, C	T	R	Decoction drunk	[15]	Kenya [29], Mozambique [30]
	*Mangifera indica* L.	Mwembe (Morogoro and Tanga)	C	T	L, B, R	Decoction drunk	[14, 15]	Brazil [31], Togo [23], South Africa [22], Nigeria [25], Rwanda [32], Zimbabwe [33]
	*Sclerocarya birrea* subsp*. caffra* (Sond.) Kokwaro	Mng'ongo (Tanga) Mumbu (Dar es Salaam) Bankuti (Pwani)	W, C	T	B	Infusion drunk	[15]	South Africa [22]

Apiaceae	*Steganotaenia araliacea* Hochst.	Semangube (Tanga)	W, C	T	B	Decoction drunk	[34]	Not found

Apocynaceae	*Strophanthus eminii* Asch. ex Pax	Musungululu (Tabora)	W	T	R, B	ns	[35]	Not found

Asparagaceae	*Dracaena steudneri* Engl.	Mgorogoro (Kagera)	C	T	L	Leaves' ashes mixed with soda ash and then licked	[36, 37]	Not found

Asteraceae	*Helianthus annuus* L.	Alizeti (Morogoro)	C	H	L	Concoction drunk	[14]	Brazil [31]
	*Psiadia punctulata* Vatke	Endimii (Arusha)	W	S	L	ns	[38]	South Africa [22]

Bignoniaceae	*Kigelia africana* (Lam.) Benth.	Mangaffi (Manyara)	W	T	ns	ns	[39]	Nigeria [24, 25]

Canellaceae	*Warburgia salutaris* (G.Bertol.) Chiov.	Musokonoi (Tabora)	C	T	B, R	ns	[40]	South Africa [22], Kenya [41]

Cannabaceae	*Trema orientalis* (L.) Blume	Msasa (Morogoro)	W, C	S	L	Decoction drunk	[38, 42, 43]	Zimbabwe [44]

Capparaceae	*Capparis tomentosa* Lam.	Mtungulang'osa (Pwani)	W	S	L	ns	[26]	Rwanda [32]
	*Maerua kirkii* (Oliv.) F.White	Msaka (Pwani)	W	S	R	Decoction drunk	[26]	Not found
	*Maerua triphylla* A.Rich.	Kilialia (Morogoro)	W	H	L	ns	[19]	Not found

Celastraceae	*Catha edulis* (Vahl) Endl.	Warfi (Manyara)	W	S	ns	Chewing; oral	[39]	Kenya [45], Ethiopia [46]

Combretaceae	*Combretum pentagonum* M.A.Lawson	Mwiza (Pwani)	W	T	B	Decoction drunk	[26]	Not found
	*Terminalia kilimandscharica* Engl.	Mkulyungu (Mtwara)	W	T	B	Decoction drunk	[47]	Not found

Cucurbitaceae	*Momordica calantha* Gilg	Fuiza (Tanga)	C	H	L, B	Leaves and bark are powdered, then mixed with honey, and taken orally	[47]	Not found

Euphorbiaceae	*Acalypha fruticosa* Forssk.	Mpasua Jabari (Kigoma)	W	S	R, L	Decoction drunk	[17]	Not found
	*Croton pseudopulchellus* Pax	Mkombechi (Tanga) Mkwati (Pwani)	W, C	T	R	Decoction; drunk	[16, 48]	Not found
	*Euphorbia hirta* L.	Mtanze (Pwani) Mziwaziwa (Dar es Salaam)	W	H	L	Leaves are pounded with water, and the juice is drunk	[16]	Togo [23], Nigeria [24, 49]
	*Synadenium glaucescens* Pax *Syn: Euphorbia neoglaucescens* Bruyns	Mwasa/Mgi (Kilimanjaro) Mluwa (Tanga and Pwani)	W	S	L	Leaf decoction is mixed with lime juice, baking soda and honey; then drunk	[16]	Not found

Fabaceae	*Abrus precatorius* L.	Mturuturu (Kigoma) Mwangaruchi (Tanga) Lufambo (Kilimanjaro) Ufambo (Pwani)	W, C	H	Fr, L, R	Powdered leaf or fruit decoction is mixed with honey or sap and taken orally. The roots are pounded, soaked in coconut juice, and then chewed	[17, 34, 50]	Togo [23], Nigeria [24, 25], Kenya [51]
	*Albizia anthelmintica* (A.Rich.) Brongn.	Mfuleta (Morogoro)	W	T	B	ns	[19]	Not found
	*Cassia absus* L. Syn: *Chamaecrista absus* (L.) H.S.Irwin & Barneby	Mlutulutu (Dar es Salaam and Pwani)	C	H	L	Decoction drunk	[15]	Togo [23], Nigeria [52]
	*Cassia mimosoides* L. Syn: Chamaecrista mimosoides (L.) Greene	Lusangalala (Morogoro)	W	H	R, B	Decoction drunk	[14]	
	*Dichrostachys cinerea* R.Vig. Syn: *Alantsilodendron pilosum* Villiers	Mkulagembe (Pwani and Dar es Salaam)	W	T	R	Decoction drunk	[50]	Sudan [53]
	*Indigofera lupatana* Baker f.	Fagio (Dar es Salaam and Pwani)	W	S	L	Fresh leaves are pounded in water; the liquid is mixed with lemon juice and drunk	[50]	Not found
	*Senegalia polyacantha* (Willd.) Seigler & Ebinger	Mgunga (Tanga and Pwani)	W	T	R	Roots boiled with soda ash, and the decoction is drunk	[48, 50]	Sudan [54]
	*Tephrosia purpurea* (L.) Pers.	Namkundi (Pwani)	W, C	H	R	Decoction drunk	[50]	Mauritius [55]
	*Vachellia nilotica* (L.) P.J.H.Hurter & Mabb.	Mugulunga (Tabora)	W, C	T	R	ns	[35]	Nigeria [24]

Lamiaceae	*Leonotis nepetifolia* (L.) R.Br.	Kivumbasi (Kigoma and Kagera)	W, C	H	Fl	Powdered flowers are added to porridge or tea and taken orally	[17, 18]	Brazil [31]
	*Ocimum basilicum* L.	Mrehani (Iringa) Luvumbapuku (Pwani) Kivumbasi kidogo (Dar es Salaam)	C	H	R	Infusion drunk	[16]	Brazil [31], Togo [23]
	*Premna chrysoclada* (Bojer) Gürke	Mtulavnha (Pwani)	W	S	L	Decoction drunk	[56]	China and Tropical Asia [57]
	*Vitex buchananii* Baker ex Gürke	Mpinga (Pwani)	W	T	R	Decoction drunk	[56]	Not found

Linderniaceae	*Lindernia insularis* Skan Syn: *Crepidorhopalon rupestris* (Engl.) Eb.Fisch.	Kikulangumbi (Kilimanjaro)	W	H	Whp	Decoction drunk	[56]	Not found

Malvaceae	*Dombeya shupangae* K.Schum	Musubu (Kigoma)	W	T	L	Decoction	[17]	Not found
	*Grewia villosa* Willd.	Mangura (Kilimanjaro)	W	S	R	Decoction drunk	[56]	Not found

Melastomataceae	*Dissotis rotundifolia* (Sm.) Triana Syn: *Heterotis rotundifolia* (Sm.) Jacq.-Fél.	Kinzasu (Morogoro)	W	H	R, L	Decoction; oral	[14]	Not found

Moraceae	*Ficus exasperata* Roxb. Syn: *Ficus ampelos* Burm.f.	Msasa (Morogoro and Dar es Salaam) Mshasha (Kilimanjaro)	W	T	R	Decoction drunk	[50]	Ivory Coast [58]

Moringaceae	*Moringa oleifera* Lam.	Mlonge (Morogoro)	C	T	L	ns	[19]	Togo [23], Nigeria [52], Kenya [41]

Musaceae	*Musa paradisiaca* L.	Idoke (Tabora)	C	H	Fl	ns	[35]	Brazil [31], Togo [23], Nigeria [24]

Phyllanthaceae	*Bridelia cathartica* Bertol.	Kamembe (Kigoma) Kipindile kwima (Tanga) Mnamanama (Pwani)	W	S	L	Infusion of leaves or decoction of root bark drunk	[16, 17]	Not found

Rhamnaceae	*Ziziphus mucronata* Willd.	Mgagawe (Tanga)	W	T	R	Roots are boiled with chicken, and the decoction is drunk	[34]	South Africa [22]

Rubiaceae	*Catunaregam nilotica* (Stapf) Tirveng.	Mdasha (Tanga)	W	S	R	Decoction or infusion drunk	[34, 59]	Not found
	*Catunaregam spinosa* (Thunb.) Tirveng.	Mtutuma (Pwani) Mwachanguku (Mwanza)	W	S	R	Decoction drunk	[59]	India [60]
	*Crossopteryx febrifuga* (Afzel. ex G.Don) Benth.	Msikosiko (Tanga)	W	T	B	Powder of a stem and root bark mixture is mixed with porridge and taken orally	[34, 59]	Not found
	*Gardenia ternifolia* Schumach. & Thonn.	Kilimandembo (Pwani) Mlipu (Kagera)	W	S	R	Dried powdered roots are mixed with porridge and drunk	[59]	Benin [61],
	*Vangueria madagascariensis* J.F.Gmel.	Mdaria (Kilimanjaro) Kamuepue (Pwani) Mvilu (Tanga)	W	T	B	Decoction drunk	[59]	Not found

Rutaceae	*Zanthoxylum chalybeum* Engl.	Mulungulungu (Tabora) Mhunungu (Dar es Salaam) Mkiti (Tanga) Namavele (Pwani) Msele (Kilimanjaro)	C	T	L, B	Dried powdered parts are added to porridge or tea and drunk	[40, 59]	Rwanda [32]
	*Citrus aurantiifolia* (Christm.) Swingle	Mudimu (Tabora)	C	T	L	ns	[35]	Togo [23], Indonesia [62]
	*Vepris eugeniifolia* (Engl.) I.Verd.	Mhombo/Mpombo (Tanga)	W, C	S	R, L	Decoction of the root with roots of *Suregada zanzibariensis* Baill. is drunk	[34]	Not found
	*Harrisonia abyssinica* Oliv.	Mkusu (Tanga and Pwani)	W	S	L	Infusion of the leaves mixed with lemon juice is drunk	[56]	Kenya [41]

Salicaceae	*Flacourtia indica* (Burm.f.) Merr.	Mgola (Pwani) Mhuli (Mwanza) Mgovigovi (Dar es Salaam)	W, C	T	L	ns	[16]	Pakistan [63]

Santalaceae	*Osyris wightiana* Wall. ex Wight Syn: *Osyris lanceolata* Hochst. & Steud.	Lasesei (Morogoro)	W	S	R	ns	[64]	South Africa [22]

Sapindaceae	*Allophylus rubifolius* (Hochst. ex A.Rich.) Engl.	Msempelele (Pwani) Mhecha (Ruvuma) Mkongodeka (Tanga)	W	S	L	Decoction drunk	[59]	Not found
	*Zanha africana* (Radlk.) Exell	Daalusamo (Manyara)	W	T	R, B	Dry powdered parts are mixed with porridge or tea and taken orally	[65]	Zimbabwe [33, 66]

Smilacaceae	*Smilax kraussiana* Meisn. Syn: *Smilax anceps* Willd	Rutamwa (Tanga)	W	H	R, L	Decoction drunk	[56]	Not found

Solanaceae	*Withania somnifera* (L.) Dunal	Olesayiet (Arusha)	W	S	R, L	ns	[38]	South Africa [22], Ethiopia [67]. India [68]

Zygophyllaceae	*Balanites aegyptiaca* (L.) Delile	Liluguyu (Mara) Nyuguyu (Morogoro)	W, C	T	B, L	Stem bark is macerated in warm water; then the extract is drunk	[19, 20]	Togo [23], India [69, 70]

So—source, C—cultivation, W—wild, LF—life form, T—tree, S—shrub, H—herb, PU—part used, L—leaf, R—root, B—bark, Fl—flower, Fr—fruit, Whp—whole plant, MoP—mode of preparation, MoA—route of administration, and ns—not specified.

**Table 2:** *In vivo* antiasthmatic activity of some of the traditional medicinal plants used in Tanzania.

Species name	Plant part	Extract/fraction/isolated compound	Dose	Experimental model of asthma	Animal model	References
*Abrus precatorius* L.	Leaves	Ethanol extract	100, 125, 150 mg/kg	Clonidine-induced catalepsy	Mice	[90]

*Acalypha fruticosa* Forssk.	Leaves	Ethanol extract	100 *µ*g/ml	His-induced	Goat tracheal chain, mice, rats	[91]

*Allium sativum* L.	Bulb	Aqueous extract	80 mg/kg	*Dermatophagoides pteronyssinus*-induced	Mice	[92]

*Balanites aegyptiaca* (L.) Delile	Fruit	n-Butanolic fraction	50, 100 and 200 mg/kg	His-induced	Guinea pigs	[70]

*Catha edulis* (Vahl) Endl.	Aerial parts	Cathine and cathinone	100–300 mg/kg	OVA-sensitized	Swiss mice, Wistar rats	[93, 94]

*Euphorbia hirta* L.	Leaf	Ethanol extract	100 *µ*g/100 *µ*l, and 200 *µ*g/100 *µ*l	Neonatal-induced	Rats	[95]
	Leaf	Ethanolic extract	500 mg/kg	His-induced	Guinea pig	[96]

*Helianthus annuus* L.	Seeds	Aqueous extract	50 *μ*g/mL	OVA-sensitized	Mice	[97]

*Leonotis nepetifolia* (L.) R.Br.	Stem	Hydroalcoholic and tea extracts	1000–3000 *µ*g/L	OVA-sensitized	Wistar rats, guinea pig	[98]

*Mangifera indica* L.	Bark	Aqueous extract, mangiferin	50, 100, 250 mg/kg (extract) and 50 mg/kg (mangiferin)	OVA-sensitized	BALB/c mice	[11]

*Moringa oleifera* Lam.	Leaf	Methanolic extract	250 mg/kg and 500 mg/kg	OVA-sensitized	Guinea pigs	[99]
	Leaf	Aqueous extract	3.9 mg/day	OVA-sensitized	BALB/c mice	[100]

*Musa paradisiaca* L.	Flower	Hydroalcoholic extract	200 and 400 mg/kg (guinea pigs), 500, 750, and 1000 *μ*g/ml (rats), and 500, 750, and 1000 *μ*g/ml (guinea pig trachea)	His-induced, ACh-induced	Guinea pigs, mice, isolated guinea pig trachea	[101, 102]

*Ocimum basilicum* L.	Leaf	Hydroethanolic extract	0.75, 1.50 and 3.00 mg/mL	OVA-sensitized	Wistar rats	[103]
	Sementes	*In natura*	5 g (5% concentration)	OVA-sensitized	BALB/c mice	[104]

*Tephrosia purpurea* (L.) Pers.	Aerial parts	Ethanolic extract	100 *µ*g/ml	Erythrocytes-induced	Rat mast cell	[105]

*Withania somnifera* (L.) Dunal	Root	Aqueous extract	200 or 400 mg/kg	OVA-induced	Wistar albino rats	[106]

## Data Availability

The information used to support the findings of this study is available from the corresponding author upon request.
